# Hepatic epithelioid hemangioendothelioma: case series of a rare vascular tumor mimicking metastases

**DOI:** 10.1186/s13000-020-01039-2

**Published:** 2020-09-25

**Authors:** Nasir Ud Din, Shabina Rahim, Tamana Asghari, Jamshid Abdul-Ghafar, Zubair Ahmad

**Affiliations:** 1 Section of Histopathology, Department of Pathology and Laboratory Medicine, Aga Khan University Hospital, Karachi, Pakistan; 2 Department of Pathology and Clinical Laboratory, French Medical Institute for Mothers and Children (FMIC), Kabul, Afghanistan

**Keywords:** Hepatic, Epithelioid hemangioendothelioma, Multifocal, ERG, CD34, CD31

## Abstract

**Background:**

Hepatic epithelioid hemangioendothelioma is an extremely rare malignant vascular tumor which is often multifocal and, in many cases, discovered incidentally. Here, we describe the clinicopathological features of hepatic epithelioid hemangioendothelioma cases seen in our practice and present a detailed review of the published literature.

**Methods:**

All cases of hepatic epithelioid hemangioendothelioma diagnosed in Department of Pathology and Laboratory Medicine, Aga Khan University Hospital between January 1, 2006 and December 31, 2019 were included in the study. Slides were reviewed and follow up was obtained.

**Results:**

Seven cases were reported during the study period. There were 4 females and 3 males. Age range was 20 to 77 years, mean age was 45 years. Three patients presented with right upper abdominal pain; 1 patient presented with jaundice while 3 patients were asymptomatic. In all 7 cases, lesions were identified on imaging studies. In 5 cases, liver lesions were multifocal. Clinical differential diagnosis in all cases was metastatic carcinoma and multifocal hepatocellular carcinoma. Liver function tests were normal in 5 cases. In 1 patient, tumor had already metastasized to the right lung. On histological examination of liver core biopsies performed in all 7 cases, classic histological features of epithelioid hemangioendothelioma were seen. Tumor cells expressed positivity for vascular markers (CD 34, CD31 and ERG) and were negative for cytokeratins, Hep par 1 and Glypican 3. Surgical resection was not performed in any of the 7 cases and all patients were treated by chemotherapy. Follow up was available in 5 cases. Of these, 3 patients died of disease and another patient was alive with metastases in both lungs, omentum and colon.

**Conclusion:**

Clinicopathological features of the 7 cases in our series and detailed review of published literature is presented. Prognosis was bad in our cases most likely due to fact that surgical resection could not be performed in any of the cases owing to lack of surgical expertise for liver tumor surgery in most parts of the country.

## Background

Hepatic epithelioid hemangioendothelioma (EHE) is a very rare malignant vascular tumor composed of epithelioid and histiocytoid endothelial cells in a myxohyaline or fibrous stroma. It may also occur in the lungs, soft tissue and bone. Primary hepatic hemangioendothelioma was first reported by Ishak et al. in 1984 [1]. Over 200 cases have so far been reported. In the liver, over 75% cases are multifocal often involving the right and left lobes of the liver. It is often discovered as an incidental finding on radiological examination but sometimes may present with abdominal pain, weight loss or ascites. Radiological findings are variable and on histological examination, the tumor may be mistaken for hepatocellular carcinoma (HCC) or metastatic carcinoma, cholangiocarcinoma, or angiosarcoma. The tumor cells are occasionally positive for cytokeratins (CKs) and express vascular markers such as CD31, CD34, and factor VIII related antigen. These tumors most commonly show a translocation of t(1; 3) (p36.3; q 25) resulting in WWTR1 – CAMTA1 fusion. YAP 1 –TFE3 fusions have also been identified. These tumors have a variable clinical course; some patients have progressive disease while others have more indolent and stable course. Owing to their rarity, these tumors may be misdiagnosed as HCC, metastatic lesions or angiosarcoma. It needs to be emphasized that owing to the rarity of these tumors, there are still no consensus treatment guidelines or protocols and these tumors are usually treated by radical surgical resection or liver transplant and/ or chemotherapy [2–7].

The aim of the present study is to present the clinicopathological features of cases of primary hepatic EHE diagnosed in our practice and to present a detailed review of the published literature on these rare neoplasms.

## Material and methods

The surgical pathology files of the Section of Histopathology, Department of Pathology and Laboratory Medicine, Aga Khan University Hospital (AKUH), Karachi were searched for cases of hepatic EHEs. All cases diagnosed between January 1, 2006 and December 31, 2019 were included in the study. Slides of all cases were retrieved and reviewed by the two principal authors (NU and ZA). Clinical and pathological features of all cases were described. Clinical follow up was obtained from the patients or family members.

## Results

**Fig. 1:**
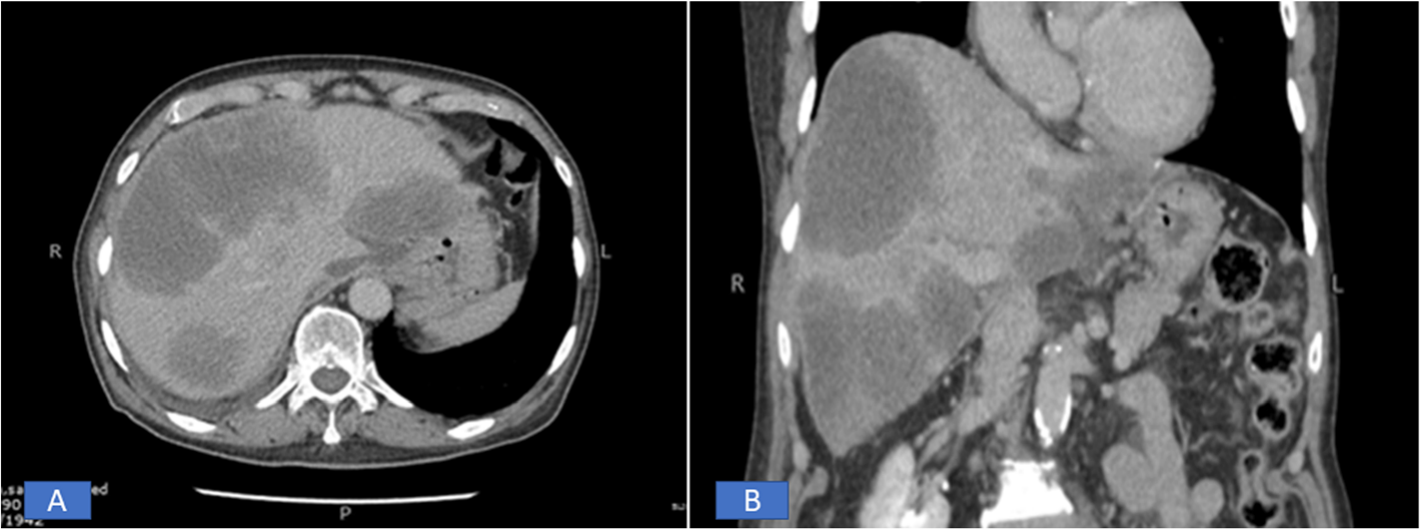
**a-b**. CT scan images of transverse and coronal section showing multiple large hypodense deposits of varying sizes, in both lobes of the liver. Radiologically, these were interpreted as most likely representing metastatic deposits

**Fig. 2:**
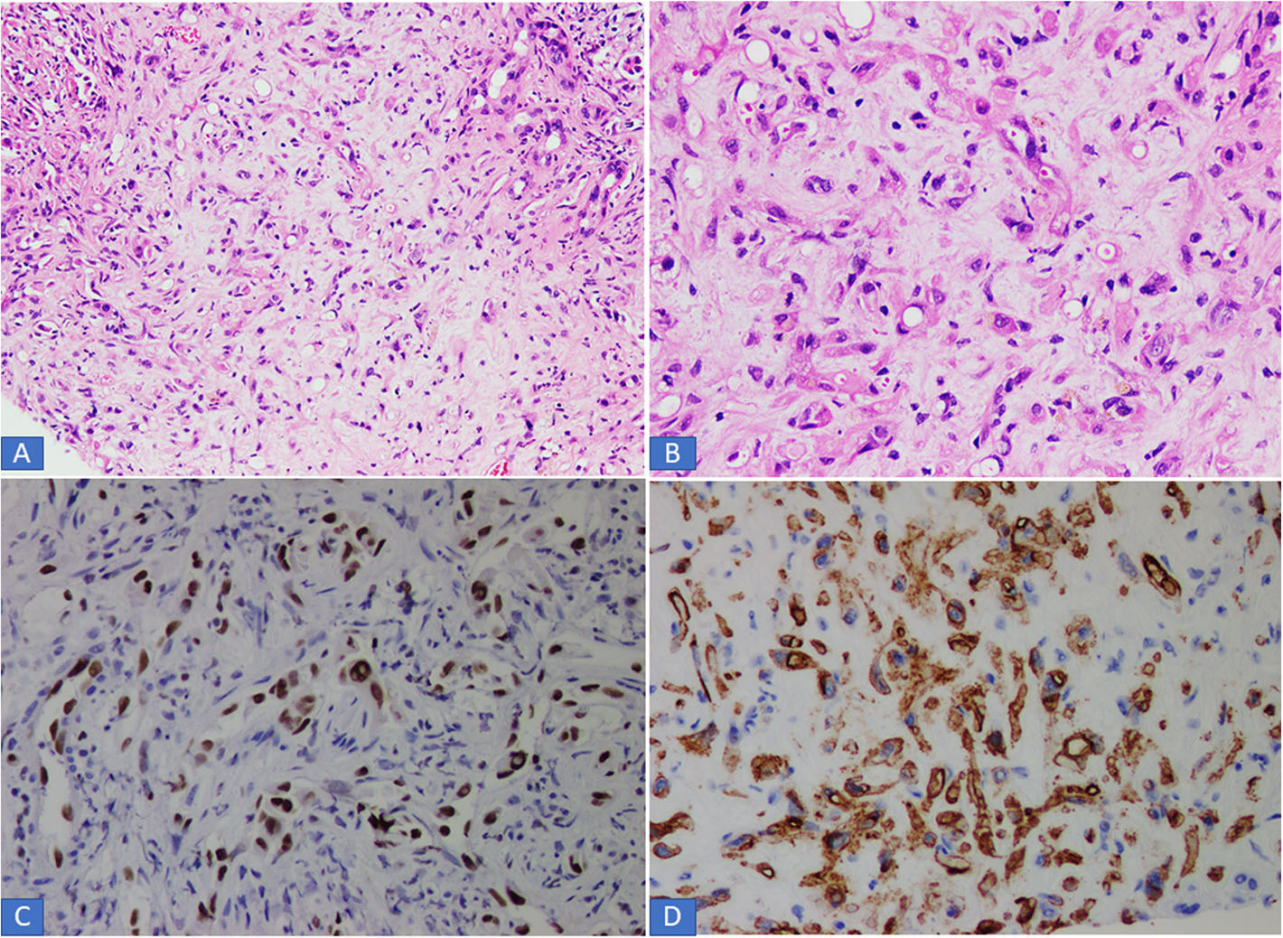
**a-b**. Intermediate and high-power examination of hepatic EHE. The tumors cells are present in a myxohyaline stroma. Cords of epithelioid cells are seen exhibiting cytoplasmic vacuoles and hyperchromatic nuclei. The tumor cells are positive for ERG **c** and CD34 **d** immunohistochemical stains

**Fig. 3:**
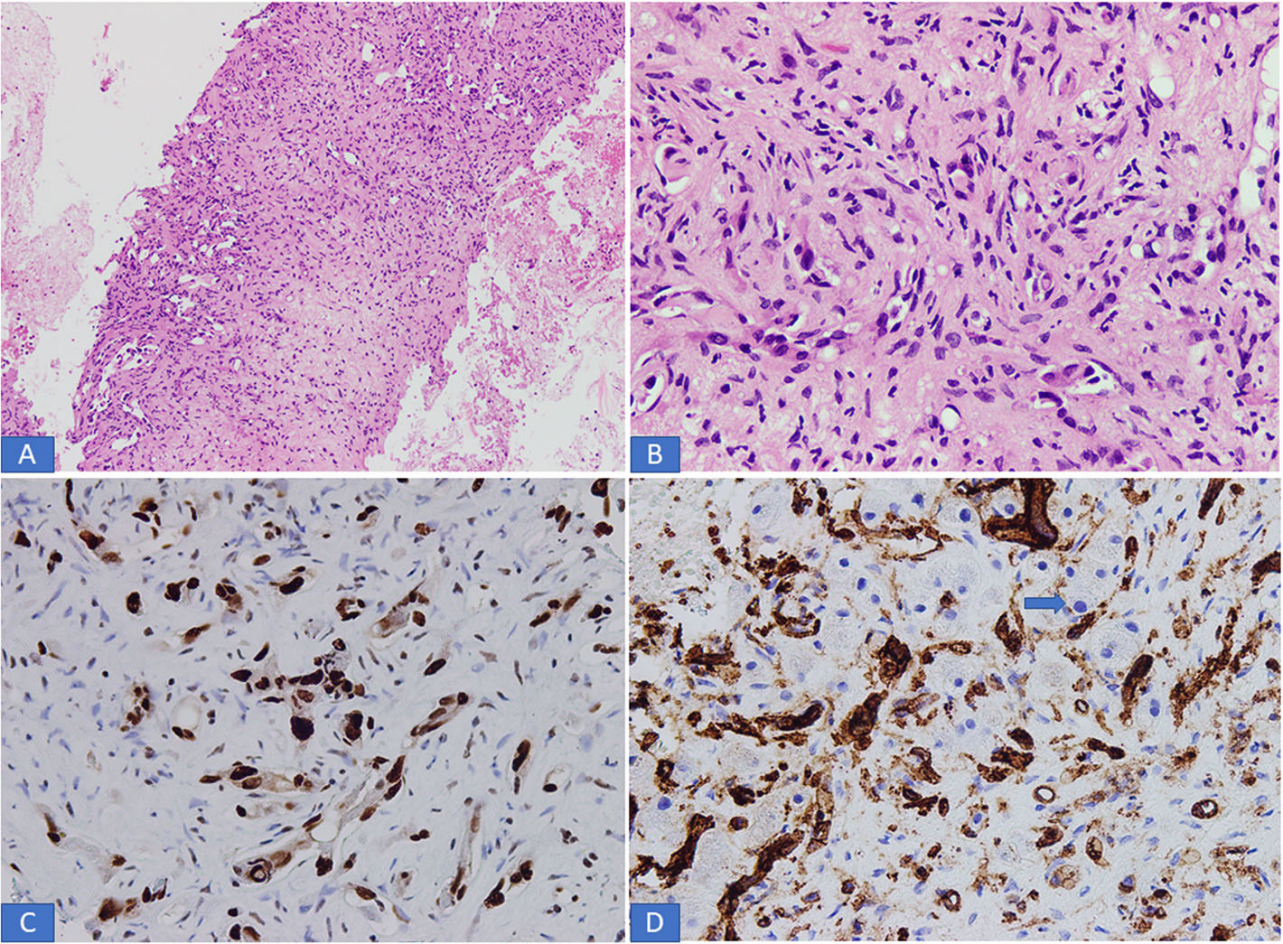
**a-b**. Low and high-power examination of hepatic EHE. Cords of epithelioid cells having cytoplasmic vacuoles and hyperchromatic nuclei. **c** Nuclear positivity for ERG. **d** CD31 positivity in tumor. The residual hepatocytes are negative for CD31 (arrow)

A total of 7 hepatic EHE were reported during the study period. Of the 7 patients, 4 (57.1%) were females and 3 (42.9%) were males. Ages of the 7 patients ranged from 20 to 77 years with mean and median age of 45 and 39 years, respectively. Out of the 7 patients, 3 (42.9%) presented with right upper abdominal pain, another patient presented with jaundice. The remaining 3 patients were asymptomatic. In all 7 patients, the lesion/s were identified on imaging studies including abdominal ultrasound, computed tomography (CT) scan and /or Magnetic resonance imaging (MRI). In 5 of the 7 patients (71.4%), the liver lesions were multifocal (multiple small nodules) in the right and left lobes of the liver (Fig. 1a, b). Clinical suspicion of metastatic tumor and multifocal HCC was raised in these cases. In the remaining two cases, single mass was seen on imaging studies and clinical suspicion of HCC was raised. In 1 of the 5 cases with multifocal lesions, concomitant multiple lesions were noted in right lung as well. Liver function tests (LFTs) were normal in 5 patients and only slightly abnormal in the other 2 cases manifesting as mild increase in serum bilirubin and aspartate and alanine aminotransferase (AST and ALT) levels. All 7 cases were received as core biopsies from the liver lesion/s and specimen comprised of single or multiple cores of greyish white tissue measuring 0.5 cm to 1.5 cm in aggregate. The cores in all 7 cases were entirely submitted for histopathological examination which showed liver parenchyma infiltrated by variably cellular neoplastic proliferation of spindled to epithelioid cells singly, in cords and nests, forming intracytoplasmic vascular lumina/vacuolations. The cells were epithelioid with abundant pale to eosinophilic cytoplasm and indistinct cell boundaries. Intraluminal tufting of cells was noted in the vascular lumina (Figs. 2 and 3 a, b). The nuclei were mild to moderately pleomorphic and hyperchromatic. In 2 cases, few intranuclear inclusions were identified. Invasion of the liver sinusoids was seen in 2 cases. All 7 cases showed only rare mitoses and significant mitotic activity was not seen. No necrosis was identified in any of the 7 cases. On immunohistochemistry (IHC), all 7 cases expressed vascular markers CD31, CD34 and ERG (Figs. 2 and 3 c, d) confirming the endothelial lineage of tumor cells and were negative for epithelial markers of CK AE1/AE3, Cam 5.2, CK7 and CK 20, Glypican3 and Hep par1. In all 7 cases, final diagnosis of hepatic EHE was rendered. Follow up was available in 5 out of 7 patients. At the time of writing this manuscript, 3 of these 5 patients had died of the disease. A young female of 20 died within 6 months of diagnosis while 2 other patients died 1 year and 5 years after initial diagnosis. All 3 patients received both chemotherapy and all ultimately developed metastases in one or both lungs. Of these 3 patients, one patient had lung metastases at the time of initial diagnosis. The remaining 2 patients in whom follow up was available were alive at the time of writing 18 months and 3 months after initial diagnosis. Both had received multiple cycles of chemotherapy and the former (a 77-year-old male) had already developed metastases in both lungs, omentum and colon. The other patient (with a 3 month follow up period) has not so far developed any recurrence or metastases. The results of follow up are shown in Table 1.

**Table 1:** Clinical data of Hepatic Hemangioendothelioma HE (*n* = 7)

S. No	Year of diagnosis	Age (years)	Gender	Location in Liver	Imaging Findings	Treatment given	Follow up
1	2006	20	F	Right Lobe	Single diffuse mass in right lobe of liver? HCC	Chemotherapy	Died of disease within 6 months of diagnosis. No known history of metastases.
2	2007	38	M	Right Lobe	Single diffuse mass in right lobe of liver? HCC	Chemotherapy	Died of disease in 2013. Received 3 cycles of chemotherapy (Carboplatin) in 2007 but did not respond. Developed lung metastasis in 2012. Histopathology of lung metastases not done.
3	2010	32	M	Both lobes of liver	Multiple masses in both lobes of liver.? Primary? Secondary	Not Known	Lost to follow up
4	2015	70	F	Both lobes of liver	Multiple masses in both lobes of liver. HCC? Metastasis	None	Died of disease in 2017. No chemotherapy given. Symptomatic treatment only. Lung metastases present on PET scan at the time of diagnosis. Histopathology of lung metastases not done.
5	2017	39	F	Both lobes of liver	Multiple masses in both lobes of liver	Not Known	Lost to follow up
6	2018	76	M	Both lobes of liver	Multiple masses in both lobes of liver	Chemotherapy	Alive with disease. On Tab Pazopanib since December 2018 to date. On PET scan -Lung metastases detected in March 2019 -Omental metastases detected in December 2019 -Colonic metastases detected in January 2020. Histopathology of metastatic lesions not done.
7	2019	45	F	Both lobes of liver	Multiple masses in both lobes of liver	None	Alive with disease. No metastasis. No chemotherapy or any other treatment given.

## Discussion

Published cases of hepatic EHEs have documented a wide range with peak incidence in middle aged adults. Mean age at presentation has ranged between 30 and 40 years. In Giardino et al’s series of 7 cases also, mean age was 47 years [8]. They are rare in children and show a slight predilection for females. However, 6 out of 7 patients in the series reported by Giardino et al. were females [8]. In our series also, a slight predilection for females was seen (4 out 7) and 3 out of 7 patients (42.9%) were asymptomatic at the time of diagnosis. Hepatic lesions vary in size from very small nodules less than 1 cm in size to large masses greater than 10 cm in diameter. Cut surface of the tumor is typically firm in consistency and white in color [2].

Various studies have reported that approximately 25 to 40% patients are asymptomatic at the time of diagnosis and hepatic lesion are often discovered incidentally. The most common complaint is right upper quadrant abdominal pain. Other symptoms usually non-specific, include ascites, weight loss, anorexia, weakness and fatigue, nausea and/ or vomiting. Hepatomegaly may be present. There may be raised alkaline phosphatase, AST and ALT. However, these may be entirely normal [3–6]. Three of our patients were asymptomatic. Three others presented with complaints of right sided upper abdominal pain while 1 patient presented with jaundice. Thus, patients were either asymptomatic (and the tumors were diagnosed incidentally on radiological examination) or showed nonspecific upper abdominal pain.

On radiological examination, lesions are multifocal in three - fourth of cases. Radiological findings are variable and may lead to confusion in diagnosis. However, according to Giardino et al., the combination of multifocal, rounded, nodular, predominantly peripheral lesions which exhibit early ring enhancement followed by the late appearance of central core of high signal and inner thick border of low signal may represent an important diagnostic feature for these lesions [8]. In our cases, large hypo dense single or multiple lesions in one or both lobes of the liver were seen and were interpreted radiologically as metastases or multifocal HCC.

Core biopsies from the lesions were performed in all 7 patients. As noted in results section, all 7 cases demonstrated the classic histological features of hepatic EHE and showed nests and cords of spindle to polygonal to epithelioid cells, with mild to moderate atypia, abundant pale to eosinophilic cytoplasm, indistinct cell boundaries and intracytoplasmic vacuolations or lumina in a fibromyxoid stroma. The lumina showed tufting of tumor cells. Sinusoidal invasion was seen in 2 cases and may be associated with necrosis. Mitotic activity was very low (< 3 mitoses per 50 High Power Fields in all 7 cases). Expression of vascular markers CD31, CD34 and ERG confirmed endothelial lineage of the tumor cells. These histological and IHC features were similar to those described first by Ishak et al. and by other authors in published literature [1–3].

Misdiagnosis occurs in approximately 60 to 80% cases according to one study [4]. The appearance can be mistaken for primary or secondary carcinomas, scirrhous variant of HCC, angiosarcoma and other undifferentiated sarcomas which are included in the histological differential diagnosis [1, 4, 6]. These tumors are distinguished from their histological mimics by a combination of histologic, IHC and molecular characteristics [3, 9]. Vascular structures of this tumor may mimic glands or tubules and lead to misdiagnosis of carcinoma. Epithelial markers are not usually expressed on IHC which coupled with the positivity for vascular markers prevents any diagnostic confusion. Although rare, hepatic EHEs may focally express CK 8 and CK 18 potentially leading to misdiagnoses as carcinoma. However, close attention to classic histological features coupled with low threshold for ordering vascular markers will avoid any potential pitfalls. Invasion of the hepatic sinusoids occurs in a number of cases and may be associated with necrosis [2, 3]. Angiosarcoma usually has more nuclear pleomorphism, atypia and mitotic activity. An important consideration in these tumors is that mitotic activity is very variable, many tumors show only rare mitoses and histological features may not be a reliable predictor of outcome. In a study of soft tissue EHE by Deyrup et al., tumors with greater than 3 mitoses/50 High Power Fields and size of greater than 3.0 cm had the worst prognosis while tumor site, cytologic atypia and presence of necrosis were not significant factors in causing an adverse outcome [10].

EHEs in liver, similar to their behavior in soft tissues, have a much better prognosis compared to angiosarcoma but may show aggressive behavior. In fact, distant metastases are seen in 20 to 30% of hepatic tumors and the number of patients dying from their disease is significant. Majority of the cases in our series showed adverse outcome with death of the patients. Complete surgical resection is paramount to achieve a favorable outcome and the lack of surgical expertise in hepatic tumor surgery in our country meant that none of the patients in our series underwent resection and were treated by chemo and radiotherapy only. This may explain the significant high mortality rates in our cases. It is also interesting that metastases and death occurred quite rapidly in our cases in spite of the fact that hepatic EHEs are not usually considered to progress so rapidly. The failure to surgically excise these tumors is most likely the main reason for such adverse outcomes. However, there are published reports in literature documenting the rapid progression of these lesions especially when multifocal (an occurrence seen in 75% of cases) [11].

As many as 90% of these tumors demonstrate a WWTR1- CAMTA1 gene fusion which results from a t(1; 3) (p36; q 25) translocation. Rarely, these tumors harbor a YAP 1- TFE3 fusion. These fusions are a characteristic feature of these tumors and are included in the World Health Organization (WHO) definition of these tumors. However, these are desirable diagnostic criteria and currently are not essential for diagnosis [2]. Studies have shown that multifocal tumors are monoclonal and represent metastatic implant of the same neoplastic clone rather than a ‘field effect’ or synchronous occurrence of multiple neoplastic clones [12].

In Mehrabi et al’s study of 434 patients published in 2006, liver transplantation was the commonest management option exercised in 44.8% patients followed by no treatment in 24.8%, chemotherapy or radiotherapy in 21% and liver resection in 9.4% patients. The 5-year survival rates were 54.5% after liver transplantation, 4.5% after no treatment, 30% after chemo or radiotherapy and 75% after liver resection [4]. Similarly, in Grotz et al’s study of 30 patients published in 2010, liver resection and liver transplant were the treatments of choice performed in 11 patients each. Liver resection was associated with a 5-year overall survival of 86% while liver transplant was associated with a 5-year survival of 73% [13]. Thus, radical surgical resection or liver transplantation are considered the methods of choice in patients with localized liver lesions. Owing to the multifocal nature of these tumors in most cases, liver transplantation is proposed as the treatment of choice in patients with multicentric tumors although liver resection has been performed more commonly [4, 14]. Liver transplantation has been considered the treatment of choice when partial hepatectomy is not feasible owing to intrahepatic dissemination. This was true in 1988 and three decades later newer reports still demonstrate that excellent results are achieved through liver resection and liver transplantation with high survival rates and high disease-free survival. Partial liver resection may not be a viable option as tumor is multilocular in many cases. Patients with these tumors are thus good candidates for liver transplantation even if extrahepatic disease is present [4, 15]. In one study, 23. 7% patients developed tumor recurrence following liver transplantation with a mean follow up period of 49 months, but extrahepatic disease and lymph node involvement did not significantly affect disease free survival [16]. Chemo and radiotherapy regimens and transcatheter arterial chemoembolization are other therapeutic options [4, 17, 18].

Unfortunately, the surgical modalities which have given excellent results in the treatment of hepatic EHE are mostly unavailable in our country and patients with these tumors are treated by chemo and radiotherapy alone resulting in poor outcomes for these patients. A recent study from China showed that surgery is the primary choice of treatment while metastases and unresectability are associated with a poor prognosis. Patients with unresectable primary tumor or distant metastases were diagnosed via biopsy and did not undergo resection. Such patients showed high mortality [19].

Another recent study by Cao et al. proposed that patients with hepatic EHE should initially undergo a period of observation during which the progression of the lesion may be assessed, and that appropriate treatment modality be selected based on this observation [20]. This approach is also supported by Rosenberg and Agulnik who outlined their treatment strategy of watch and wait versus active therapy on clinical trial. They believe that since the spectrum of disease varies widely between an indolent tumor and aggressive disease with widespread metastases, the primary focus should be to determine whether the disease will behave aggressively or not based on which it would be determined how aggressively the patient should be treated [9]. In a recent 2020 study, Noh et al. evaluated the management and prognosis of these tumors based on the Surveillance. Epidemiology and End Results (SEER) program data analysis from 1973 to 2014 and showed that no treatment was the most common management (39.2%) followed by chemotherapy only (29.7%), liver resection (17.6%) and liver transplantation (8.1%). They reported that patients who underwent surgical treatment had much higher survival rates than those who were treated non- surgically (5-year survival rates 88% versus 49%). Surgical therapy was shown to be the only independent prognostic factor for survival by multivariate analysis [21]. Similarly, a case report by Mihaylor et al. reported a patient treated with liver transplant who was doing well 10 years later with no signs of recurrence [18].

Chemotherapeutic agents used in the treatment of these lesions include vincristine, doxorubicin, thalidomide, 5- fluorouracil, interferon–alpha and monoclonal antibodies against vascular endothelial growth factors. Drugs with anti- angiogenic activity such as bevacizumab, sorafenib, thalidomide and lenalidomide have been used [3]. More recent studies have recommended further investigation to determine the role of chemotherapeutic and targeted therapies with specific focus on anti-angiogenesis as well as cytokines and immunotherapy which have all shown some promise in clinical trials [9, 22].

Through the years, these tumors continue to make up for case reports. It is also important to remember that these often incidentally recognized tumors may co–exist with other liver tumors thus complicating the patient’s management. Athanasopoulos et al. published a case report of synchronous hepatic EHE and HCC in a 58 years old male [23]. One case in our series metastasized to the omentum and colon. Nakamura et al. published a case of hepatic EHE which metastasized to the mesentery of the small intestine [24]. Shah et al. reported a case of multifocal hepatic EHE developing in the background of Budd- Chiari syndrome with secondary cirrhosis. The tumor was aggressive and within a few months showed common bile duct infiltration and vascular invasion and metastasized rapidly to the spleen and dorsolumbar vertebrae [25]. Another recent report demonstrated the first known occurrence of primary hepatic EHE in a patient with alcoholic cirrhosis of the liver [26].

## Conclusion

Clinicopathological features of the 7 cases in our series and detailed review of published literature was presented. Prognosis was bad in our cases most likely due to fact that surgical resection could not be performed in any cases owing to lack of surgical expertise for liver tumor surgery in most parts of the country. It is tragic as these tumors have a much better prognosis than hepatic angiosarcoma. Awareness about these lesions and availability of skilled hepatic surgeons is essential in order to achieve good therapeutic response and better prognosis in these patients.

## Data Availability

All data generated or analyzed during this study are included in this published article.

## Abbreviations

EHE: Epitheloid hemangioendothelioma
HCC: Hepatocellular carcinoma
AKUH: Aga Khan University Hospital
CT: Computed tomography
MRI: Magnetic resonance imaging
LFT: Liver function tests
IHC: Immunohistochemistry
WHO: World Health Organization
